# Impact of SARS-CoV-2 RBM Mutations N501Y and E484K on ACE2 Binding: A Combined Computational and Experimental Study

**DOI:** 10.3390/ijms26094064

**Published:** 2025-04-25

**Authors:** Agnieszka Rombel-Bryzek, Peicho Petkov, Elena Lilkova, Nevena Ilieva, Leandar Litov, Mariusz Kubus, Danuta Witkowska

**Affiliations:** 1 Institute of Medical Sciences, University of Opole, 45-052 Opole, Poland; 2 Faculty of Physics, Sofia University “St. Kliment Ohridski”, 1164 Sofia, Bulgaria; leandar.litov@cern.ch; 3 Institute of Information and Communication Technologies, Bulgarian Academy of Sciences, 1113 Sofia, Bulgaria; elena.lilkova@iict.bas.bg (E.L.); nevena.ilieva@iict.bas.bg (N.I.); 4 Centre of Education and Mathematics Applications, Opole University of Technology, 45-758 Opole, Poland; m.kubus@po.edu.pl; 5 Institute of Health Sciences, University of Opole, 45-060 Opole, Poland; danuta.witkowska@uni.opole.pl

**Keywords:** SARS-CoV-2, ACE2, N501Y, E484K, binding affinity, ITC, molecular dynamics

## Abstract

The SARS-CoV-2 spike receptor-binding motif is crucial for viral entry via interaction with the human ACE2 receptor. Mutations N501Y and E484K, found in several variants of concern, impact viral transmissibility and immune escape, but experimental data on their binding effects remain inconsistent. Using isothermal titration calorimetry (ITC) and molecular dynamics (MD) simulations, we analyzed the thermodynamic and structural effects of these mutations. ITC confirmed that N501Y increases ACE2 affinity by 2.2-fold, while E484K enhances binding by 5.8-fold. The Beta/Gamma variant (carrying both mutations) showed the strongest affinity, with a 15-fold increase. E484K was enthalpy-driven, while N501Y introduced entropy-driven effects, suggesting hydrophobic interactions and conformational changes. MD simulations revealed distinct binding poses, with Beta/Gamma peptides interacting with a secondary ACE2 site. A strong correlation was found between entropy contributions and hydrophobic contacts. Additionally, a convolutional neural network was used to estimate the free binding energy of these complexes. Our findings confirm that N501Y and E484K enhance ACE2 binding, with the greatest effect when combined, providing insights into SARS-CoV-2 variant evolution and potential therapeutic strategies.

## 1. Introduction

Severe acute respiratory syndrome coronavirus 2 (SARS-CoV-2) is a highly transmissible and pathogenic coronavirus responsible for the COVID-19 pandemic. Since its emergence in late 2019, the virus has undergone continuous evolution, leading to the emergence of multiple variants with distinct genetic profiles [1]. While most mutations have minimal impact, some introduce new features or, more commonly, enhance existing traits that benefit the virus, such as increased transmissibility, greater disease severity, immune evasion, reduced vaccine or therapeutic efficacy, and compromised diagnostic accuracy [2]. Based on their epidemiological significance, the World Health Organization (WHO) categorizes them as variants of concern (VOCs) and variants of interest (VOIs) [3,4,5]. Tracking these mutations provides crucial insights into viral evolution and adaptation, informing the development of effective vaccines and therapies [6].

The spike protein (S) of SARS-CoV-2 is a key structural component responsible for mediating viral entry into host cells. It consists of two subunits: S1, which contains the receptor-binding domain (RBD), and S2, which facilitates membrane fusion [7]. The interaction between the RBD and the angiotensin-converting enzyme 2 (ACE2) receptor on human cells is critical for viral attachment and internalization [8]. The RBD-ACE2 interface includes 17 amino acid residues at the spike RBD, 16 of which are found in a short segment (67 residues) known as the receptor-binding motif (RBM) [9,10]. Given its functional significance, the S-protein is a primary target for therapeutic antibodies and vaccines.

Mutations in the S-protein, particularly within the RBD, can influence viral properties such as transmissibility, infectivity, and immune evasion. Several well-characterized mutations, including N501Y, E484K, K417N, and D614G, have been shown to affect ACE2 binding affinity and resistance to neutralizing antibodies [11,12]. The RBM, which directly interacts with ACE2, harbors several of these critical mutations [13]. While mutations N501Y and E484K provide a distinct selective advantage for the virus, as shown in multiple studies [8,9], they are located relatively far apart within the RBM [14].

Among the many mutations identified in the RBM, N501Y and E484K have garnered particular attention due to their significant effects on receptor binding and immune evasion. The N501Y mutation was first detected in the B.1.1.7 (Alpha) variant and later found in other VOCs, including Beta, Gamma, and Omicron [15,16]. Several studies have reported that N501Y significantly increases binding affinity to the host cell receptor, potentially enhancing viral transmission [6,9,17,18]. Moreover, this mutation may confer a slight increase in resistance to neutralizing antibodies [13]. The E484K mutation, first identified in the B.1.351 (Beta) variant, was later detected in other variants, including P.1 (Gamma), P.2 (Zeta), B.1.525 (Eta), and B.1.526 (Iota). Studies suggest that while E484K moderately increases the RBD’s binding affinity for ACE2, this effect is less pronounced than that of the N501Y mutation [14,15,19]. Additionally, E484K is recognized as an escape mutation, enhancing the virus’s ability to evade neutralizing antibodies [20].

Despite extensive research on the effects of N501Y and E484K, the reported binding affinities and structural changes associated with these mutations vary significantly across studies. Some molecular dynamics (MD) simulations suggest a substantial increase in ACE2 binding affinity, while others report only marginal differences [11]. Similarly, experimental approaches, including surface plasmon resonance (SPR) and biolayer interferometry (BLI), have produced divergent results [13].

To address the discrepancies in the reported binding affinities and structural changes induced by specific mutations in the SARS-CoV-2 receptor-binding domain, we conducted a comprehensive study integrating both experimental approaches and theoretical simulations. By comparing not only the binding affinity but also the enthalpy of interactions, we aimed to provide a more conclusive understanding of how these mutations influence the RBD’s interaction with the human ACE2 receptor. This dual approach allows us to assess the thermodynamic parameters governing the binding process, offering insights into the molecular mechanisms underlying the effects of these mutations.

## 2. Results

**Figure 1:**
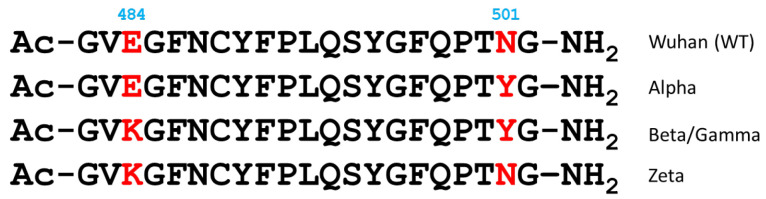
Sequences of the analyzed RBM fragments (peptides) with the single and combined mutations. The variable amino acids are shown in red.

**Figure 2:**
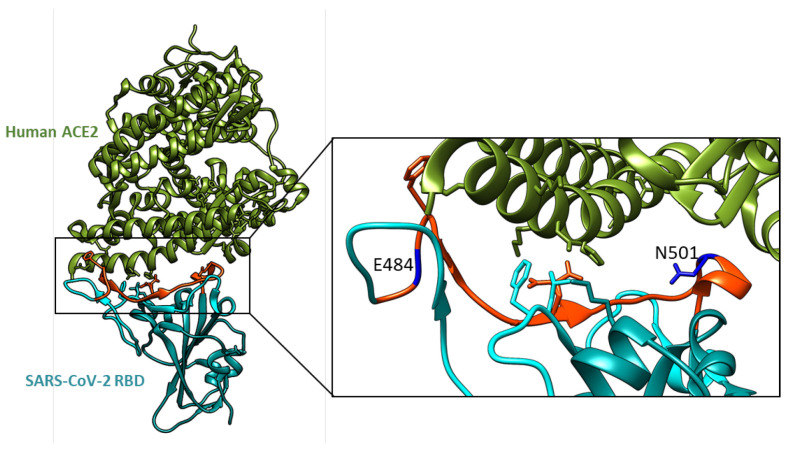
The overall structure of the SARS-CoV-2 RBD bound to the ACE2 receptor. The analyzed RBM fragment is shown in red, with the variable amino acids highlighted in blue. Visualized using UCSF Chimera [21], PDB ID: 6M0J.

The previous studies investigating the effects of mutations on SARS-CoV-2 binding to the ACE2 receptor have primarily focused on the RBD or the entire S1 subunit of the spike protein. To specifically assess the role of the N501Y and E484K mutations in ACE2 binding, we generated RBM fragments (peptides) containing one or both mutations (Figure 1). Their location in the RBD-ACE2 receptor complex is shown in Figure 2.

### 2.1. ITC Results

Isothermal titration calorimetry was employed to assess binding affinities, enthalpy, and entropy changes in the interactions between four receptor binding motif (RBM) fragments and the hACE2 receptor. The ITC data revealed that all analyzed peptides bind to ACE2 with a 1:1 stoichiometry (see Table 1). The ITC curves are shown in Supplementary Figures S1.

All mutations tested result in increased binding affinity of RBM peptides to the human ACE2 receptor (Table 1). The N501Y mutation, present in the Alpha variant, increases affinity by 2.2-fold, while the E484K mutation, found in the Zeta variant, enhances it by 5.8-fold. Notably, the RBM fragment containing both mutations exhibits the highest affinity for hACE2, with a remarkable 15-fold increase. These co-occurring mutations are present in the RBM of the Beta and Gamma variants.

**Figure 3:**
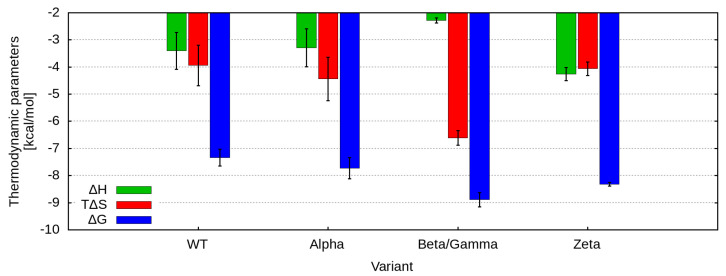
Thermodynamic signatures obtained from ITC measurements, detailing the binding interactions between ACE2 and the analyzed RBM fragments of five SARS-CoV-2 variants at pH 7.4 and 25 °C. The Gibbs free energy change (ΔG) is represented in blue, the enthalpy change (ΔH) in green, and the entropy term (−TΔS) in red, with all values expressed in kcal/mol.

All four systems exhibit binding behavior influenced by both enthalpic and entropic contributions, as detailed in Table 1 and illustrated in Figure 3. For the Zeta peptide, which carries the single E484K mutation, the enthalpic and entropic contributions are of comparable magnitude within the experimental error. Conversely, the interaction between ACE2 and the peptide containing both the N501Y and E484K mutations (the Beta/Gamma peptide) demonstrates a significant entropic contribution relative to the enthalpic component. This observation suggests alterations in hydrophobic interactions or conformational dynamics that may play a crucial role in the binding process.

### 2.2. MD Results

ITC provides high-quality experimental macroscopic thermodynamic data about the binding of two molecules, but it does not reveal the molecular details underlying these energetic contributions. MD simulations, on the other hand, build a detailed microscopic model on the atomic level of the underlying interactions, governing the binding process. To gain molecular-level insights into our experimental findings, we conducted MD simulations to study the interactions between each peptide fragment (WT, Alpha, Beta/Gamma, and Zeta) and the glycosylated hACE2 receptor. To closely resemble the experimental setup, each simulation began with the free, unrestrained peptide positioned approximately 2 nm from the receptor surface. Two independent simulations, each lasting 400 nanoseconds, were performed for each peptide, starting from distinct initial configurations. Given that ACE2 is a glycoprotein and its glycosylation influences SARS-CoV-2 S protein binding [22], a glycosylated receptor model was used in the MD simulations.

**Figure 4:**
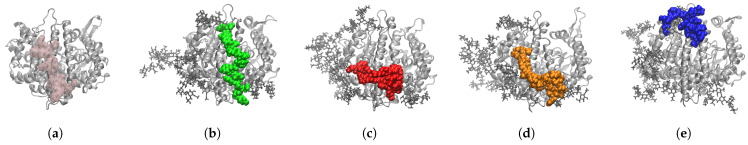
Binding of the peptides to glycosylated hACE2. (**a**) The surface of the hACE2 enzyme at the SARS-CoV-2 spike–ACE2 binding interface. The enzyme is depicted in silver cartoons and the binding surface in a pink wireframe. (**a**–**e**) Binding poses of the WT (**b**), Alpha (**c**), Beta/Gamma (**d**), and Zeta (**e**) RBM peptide fragments. The peptides are depicted in green, red, orange, and blue Van der Waals surface, respectively.

The binding interface of the hACE2 enzyme with the SARS-CoV-2 spike protein was determined from experimental structures of the complex (PDB IDs 2AJF, 3KBH, 3SCI, 3SCJ, 6LZG, 6M0J, 6VW1, 7DMU, 7DRV, 7EFP, 7EFR, 7EKC, 7EKE, 7EKF, 7EKG, 7EKH, 7L0N, 7LO4, 7NXC, 7P19, 7P8I, 7P8J, 7PKI, 7RPV, 7SN0, 7TN0, 7U0N, 7UFK, 7VIB, 7WBP, 7WBQ, 7WHH, 7WNM, 7XAZ, 7XB0, 7XB1, 7XWA, 7ZF7, 8ASY, 8DF5, 8H5C, 8IF2, 8SPH, 8SPI) and is shown in Figure 4a.

**Figure 5:**
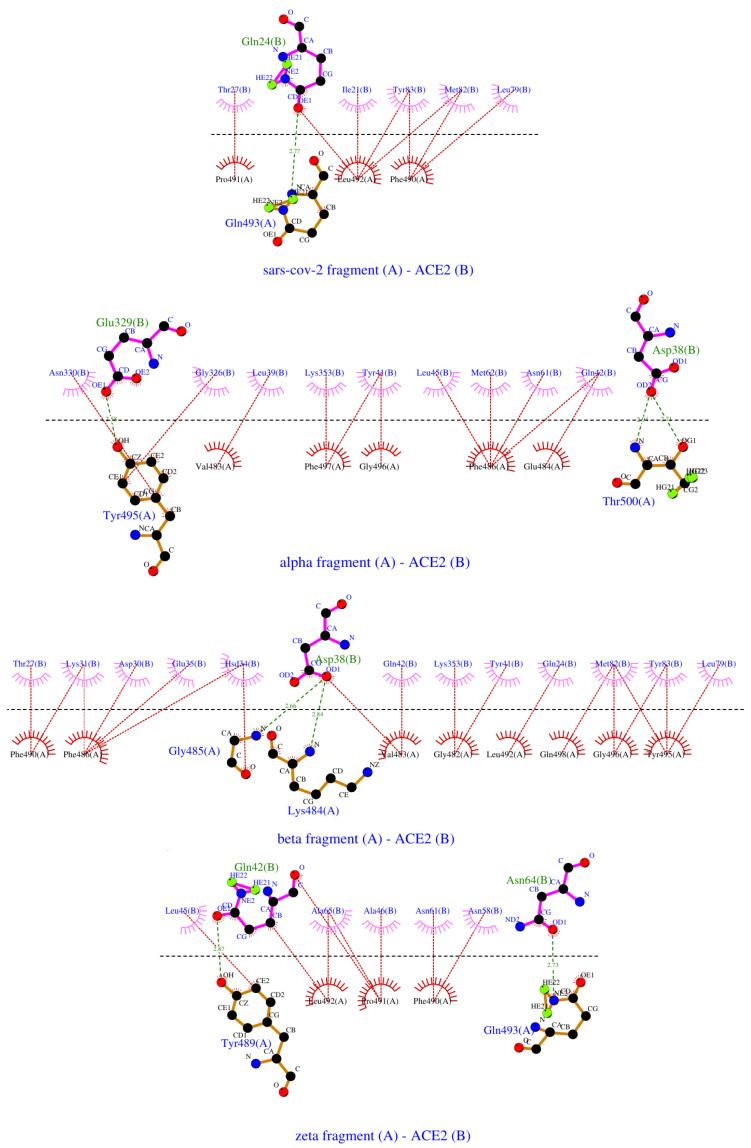
2D LigPlot representation of peptide–ACE2 interactions. Covalent bonds are presented in solid brown lines in the RBM fragments and solid purple lines in the ACE2 receptor. Intermolecular H-bonds are shown in dashed green lines, with participating amino acids noted in green in the peptides and blue in the receptor. Hydrophobic contacts are depicted with red dashed lines and arcs with radiating lines in magenta or red for the ACE2 and the peptides, and the relevant amino acids are denoted in blue and black, respectively.

We observed that all peptides recognize the ACE2 receptor domain responsible for binding the SARS-CoV-2 spike protein. While all peptides bound to the same general region of ACE2, distinct differences in binding poses were observed among the four variants (Figure 4). In particular, the Beta/Gamma peptide fragment engaged a secondary binding site on the receptor, involving residues K^313^–N^321^, H^417^–I^421^, T^548^–R^559^, and the glycans at N^90^, N^322^, and N^546^. Occasionally, the Zeta peptide also interacted near this site, targeting residues T^324^Q^325^, K^353^–R^357^, A^386^A^387^, N^556^–V^574^, and the same glycans N90, N322, and N546. An overview of the generated conformational ensembles for each system is available in Supplementary Figure S2 in the Supplementary Material. A summary of the interactions between the various peptide fragments and ACE2, based on the final conformations, is shown in Figure 5.

**Figure 6:**
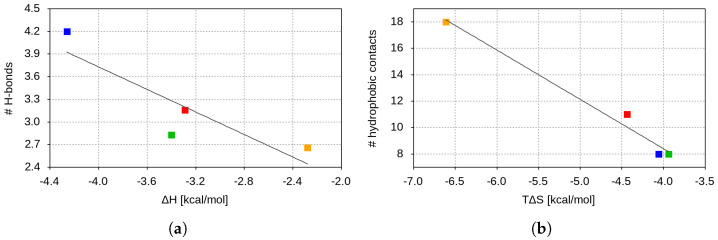
Correlation between (**a**) the number of peptide–hACE2 hydrophobic contacts and the experimentally determined entropic contribution to the binding free energy and (**b**) the number of H-bonds between the peptides and the glycosylated hACE2 and the experimentally measured enthalpy change upon binding. The values for the WT, Alpha, Beta/Gamma, and Zeta RBM fragments are shown in green, red, orange, and blue squares, respectively.

Our in silico data suggest a strong correlation between the experimentally measured entropy contribution to the binding free energy and the number of hydrophobic contacts between the binding partners (Figure 6a). The Beta/Gamma peptide, forming 18 hydrophobic contacts with the receptor, exhibits the largest entropy change upon binding among the studied variants, supporting our earlier observations. Additionally, this peptide adopts an alternative binding pose, further contributing to the entropic term of the binding. The Alpha fragment forms approximately 60% fewer hydrophobic contacts (eleven) with the ACE2 receptor, which aligns closely with the 67% lower entropic contribution to its binding free energy compared to the Beta/Gamma variant. The WT and Zeta peptides each form about eight hydrophobic contacts with the receptor and display similar entropic contributions. This correlation aligns with the previous studies, indicating that the number of favorable contacts between binding partners can directly influence the total configurational entropy.

Based on the LigPlot analysis, no correlation between binding enthalpies and the number of hydrogen bonds formed between the peptide and the receptor could be identified, which is to be expected due to the limited number of amino acid residues involved in hydrogen bonding in both binding partners. However, this analysis does not account for hydrogen bonds formed between the peptide fragments and the glycans of hACE2. With these interactions taken into account, a strong correlation emerges between the experimentally measured enthalpies (as shown in Table 1) and the number of hydrogen bonds formed between the peptides and the glycosylated hACE2 (Figure 6b). This finding aligns with the previous studies, suggesting that hydrogen bonds significantly contribute to binding enthalpy, especially in interactions involving glycosylation regions.

### 2.3. CNN Predictions

A convolutional neural network (CNN) was developed to predict the binding free energy of protein–peptide complexes. The network processes a 3D spatial grid, where each voxel represents an amino acid residue, distinguishing between receptor and ligand residues. It employs two parallel convolutional pathways with max-pooling and average-pooling to extract complementary spatial features. The trained model was applied to the four investigated complexes, yielding the following predicted binding free energies:

$$\begin{array}{cc} \Delta G_{\mathrm{CNN}}\left(\mathrm{WT}\right) & =-12.2\pm 1.9\;\mathrm{kcal}/\mathrm{mol}\\ \Delta G_{\mathrm{CNN}}\left(E484K\right) & =-12.8\pm 1.9\;\mathrm{kcal}/\mathrm{mol}\\ \Delta G_{\mathrm{CNN}}\left(N501Y\right) & =-17.0\pm 1.9\;\mathrm{kcal}/\mathrm{mol}\\ \Delta G_{\mathrm{CNN}}(N501Y\;+\;E484K) & =-15.4\pm 1.9\;\mathrm{kcal}/\mathrm{mol} \end{array}$$

The CNN predictions indicate that N501Y leads to the strongest binding affinity, while E484K alone has a moderate effect. Interestingly, the double mutation (N501Y + E484K) does not follow a strictly additive trend, suggesting potential structural or energetic compensatory effects. Notably, the CNN estimations align well with our experimental findings.

### 2.4. Comparison with Other Experimental Measurements and Theoretical Predictions

**Figure 7:**
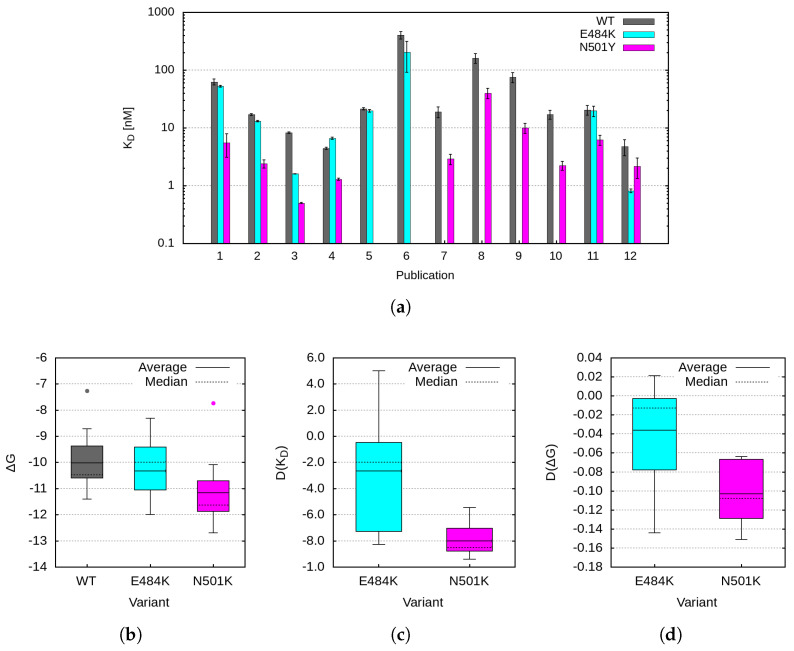
(**a**) Experimental results for K_D_ measurement as obtained in publications [14,15,17,23,24,25,26,27,28,29], and this study, numbered in the plot consecutively from 1 to 11 and listed in Supplementary Table S1; (**b**–**d**) Summaries of the distribution of the (**b**) calculated ΔG for the WT and the E484K and N501Y mutations (the dots on the box diagram correspond to our results); (**c**) D(K_D_), and (**d**) D(ΔG) for the E484K and N501Y mutations.

We identified eleven experimental data points reporting binding affinities (K_D_) for the interaction between hACE2 and the spike protein’s RBD in its wild-type form or carrying the E484K, N501Y, or combined mutations. The obtained results are presented graphically in Figure 7a. In cases where authors did not report measurement errors, an estimated error based on the typical uncertainty of the corresponding measurement methodology was assigned. These errors were then treated and propagated according to standard statistical error propagation rules. An overview of the experimental methods and conditions in each study is provided in Supplementary Table S1. The ΔG values corresponding to the respective K_D_ values are shown in Supplementary Figure S3a, whereas a box and whiskers summary is provided in Figure 7b.

As seen in Figure 7a, a significant discrepancy (>5σ) is observed among different K_D_ measurements. The primary reasons for this variation are differences in the RBD constructs used in experiments (i.e., variations in the number of amino acids included) and diversity in experimental methodologies [30]. To partially minimize the influence of experimental conditions and potential systematic errors, we used the relative change in binding affinity or binding free energy ΔG, defined as

(1) $$D\left(X\right)=\frac{X_{m}-X_{wt}}{X_{wt}}$$

where *X* represents either K_D_ or ΔG for the WT or the corresponding mutation. The obtained D(K_D_) values are plotted in Supplementary Figure S3b, with a box and whiskers summary in Figure 7c, where the observed discrepancies are significantly reduced, as expected. These data allow us to draw a definitive conclusion regarding the positive impact of the N501Y mutation on binding affinity. However, determining the effect of the E484K mutation remains challenging. The calculated mean values are

$$\begin{array}{cc} D\left(K_{D}\left(N501Y\right)\right) & =0.8\pm \;0.11\\ D\left(K_{D}\left(E484K\right)\right) & =-0.27\pm 0.41 \end{array}$$

Thus, for the E484K mutation, the relative K_D_ difference is statistically compatible with zero, suggesting no significant change in binding affinity.

Theoretical predictions [31,32,33,34] focus on binding free energy ΔG. Its logarithmic dependence on K_D_ visibly smears the diverging dissociation-coefficient results. The calculated binding free energies based on the measured K_D_ values are shown in Supplementary Figure S3a and summarized in Figure 7b. The respective mean values

$$\begin{array}{cc} \Delta G\;\left(\mathrm{WT}\right) & =-10.0\pm 1.1\;\mathrm{kcal}/\mathrm{mol},\\ \Delta G\;\left(E484K\right) & =-10.3\pm 1.1\;\mathrm{kcal}/\mathrm{mol},\\ \Delta G\;\left(N501Y\right) & =-11.2\pm 1.3\;\mathrm{kcal}/\mathrm{mol}, \end{array}$$

highlight the N501Y mutation as the strongest binder (with the most negative binding energy), while the binding energy for the E484K mutation remains comparable to that of the wild type.

The relative change in binding energy (D(ΔG)) shown in Figure 7d further supports the conclusion that the N501Y mutation significantly enhances the binding affinity of the SARS-CoV-2 spike protein to ACE2. However, a similar conclusion regarding the E484K mutation cannot be drawn from the existing data.

**Figure 8:**
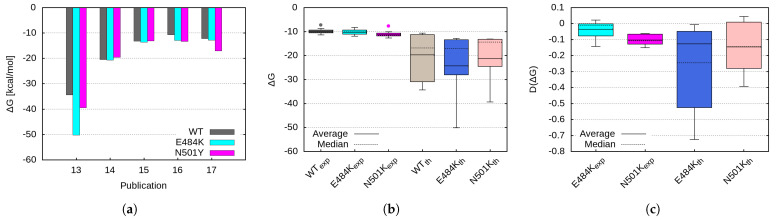
(**a**) Theoretical estimations of binding free energy (results from [31,32,33,34], with the last data point representing the CNN prediction from this study); Comparison of the theoretical estimations with experimental data for the (**b**) binding free energy and (**c**) relative change in binding free energy.

Several studies have estimated theoretically the binding free energy of the spike protein or various parts of it, bearing the investigated mutation to the ACE2 receptor [31,32,33,34]. Their results are summarized in Figure 8a, with a comparison between experimental measurements and theoretical predictions presented in Figure 8b,c. The theoretical models consistently overestimate the binding energy compared to the experimental data, except for estimations from the Prodigy server, which show relatively good agreement with the experimental results [33,34]. The binding energy predictions from our CNN model closely align with those obtained from the Prodigy server and suggest significant changes in binding affinity in the case of the N501Y mutation and the combined E484K + N501Y mutations.

As expected, our binding-energy measurements yield lower absolute values due to the smaller size of the investigated representative RBM domains, which consist of only 21 amino acids. Nevertheless, the relative change in D(ΔG) (Figure 8c) is in good agreement with other studies. Our data suggest that both mutations induce a significant increase in binding affinity, with the effect being more pronounced for the E484K mutation.

## 3. Discussion

This study aimed to elucidate the impact of the N501Y and E484K mutations in the SARS-CoV-2 spike receptor-binding motif (RBM) on its interaction with the human ACE2 receptor by combining isothermal titration calorimetry (ITC) with molecular dynamics (MD) simulations and machine-learning-based free energy prediction. Our integrative approach enabled a multi-scale understanding of binding affinity changes—from macroscopic thermodynamic signatures to atomic-level interaction patterns.

There are multiple experimental and theoretical studies on the effect of the N501Y and E484K mutations on the binding of the RBD of the S-protein to the ACE2 receptor. Their results vary significantly due to the use of different experimental techniques and conditions. ITC is the only technique that directly provides binding free energies as it measures heat changes during binding events. However, these are strongly affected by pH, ionic strength, buffer composition, and temperature. Therefore, K_D(ITC)_ and ΔG_ITC_ are difficult to compare to results from other techniques unless conditions are tightly controlled. SPR directly provides association and dissociation rate constants, but they may also be affected by temperature, buffer, and particularly surface immobilization that can alter protein conformation or sterically hinder binding, as well as sensitivity to non-specific interactions between sensor surface and analyte. BLI measurements often match SPR well despite its lower sensitivity to detection. However, similarly to SPR, BLI can diverge from ITC due to improper immobilization. Nonetheless, K_D_ or ΔG are well-defined physical quantities that characterize the equilibrium between bound and unbound species. While their measured value may vary depending on the experimental method and conditions, comparing K_D_ or ΔG values obtained by different techniques remains informative.

The E484K mutation involves the substitution of a negatively charged glutamic acid (E) at position 484 in the wild-type RBD with a positively charged lysine (K) [15,19]. In the wild-type RBD, Glu^484^ forms an ionic bond with Lys^31^ of the human ACE2 receptor. The E484K substitution disrupts this interaction but allows Lys^484^ to potentially form a new ionic bond with Glu^35^ of ACE2 [14]. The reported data on the effect of the E484K mutation on the binding affinity between the SARS-CoV-2 RBD and the hACE2 receptor diverge significantly [6,14,15,29]. Some studies utilizing SPR have reported minimal to modest increases in binding affinity, approximately 1.4- to 1.5-fold. Structural analyses suggest that the E484K mutation may disrupt an ion pair between E484 in the RBD and K31 of ACE2. This disruption allows for the formation of a new ion pair between K484 and E35 on ACE2, potentially enhancing binding affinity. This mutation was proposed to alter the charge in the flexible loop region of the RBD, facilitating a favorable interaction with E75 in ACE2, thereby increasing affinity [35]. However, the extent of these effects varies across studies, highlighting the complexity of the E484K mutation’s impact on RBD-ACE2 interactions.

The N501Y mutation involves the substitution of asparagine (N) at position 501 in the wild-type RBD, which forms a single hydrogen bond with the tyrosine (Y^41^) residue of hACE2 [9] by tyrosine (Y) [36]. Studies have demonstrated that the N501Y mutation enhances the interaction between SARS-CoV-2 and hACE2 through increased hydrophobic interactions and π-electron stacking. This substitution introduces additional π–π and π–cation interactions, further strengthening the binding affinity between the RBD and the ACE2 receptor. Additionally, the hydroxyl group of Tyr^501^ can form hydrogen bonds with Lys^353^ and Asp^38^ on ACE2, further stabilizing the complex [14,17,18,19]. However, the magnitude of this enhancement varies depending on the length of the spike protein analyzed and the assay technique used, such as surface plasmon resonance, biolayer interferometry, isothermal titration calorimetry, or microscale thermophoresis [2]. For example, biolayer interferometry measurements showed only a slight 1.3-fold increase in ACE2 binding affinity when comparing unmutated and N501Y spike protein ectodomain trimers [37]. Conversely, other studies have observed a more pronounced increase in the RBD’s affinity for ACE2 due to this mutation, with reported values ranging from 1.98- to 10.6-fold [6,14,15,17,18,25,26,27,28,29,38,39].

Consistent with our findings, a significant increase in the RBD’s affinity for ACE2 in the case of combined N501Y and E484K mutations was reported in [14,15]. This increase, which exceeds the additive effect of the individual mutations, may be attributed to positive cooperativity between the mutations or the experimental variations [14].

We found that both N501Y and E484K mutations enhance ACE2 binding, with the greatest affinity observed when both mutations are present (Beta/Gamma variant). The ITC measurements revealed a 2.2-fold increase in binding affinity for the N501Y-containing Alpha peptide and a 5.8-fold increase for the E484K-containing Zeta peptide. Strikingly, the combination of both mutations resulted in a 15-fold increase in affinity. These findings are consistent with prior studies [14,15].

By analyzing enthalpic and entropic contributions to binding, we observed that the E484K mutation promotes enthalpy-driven binding, likely due to new hydrogen bonds involving glycan sites, while the N501Y mutation contributes predominantly through entropy, likely reflecting enhanced hydrophobic interactions and increased flexibility. Our MD simulations corroborated this view by showing that the Beta/Gamma peptide formed the highest number of hydrophobic contacts and frequently adopted an alternative binding pose at a secondary ACE2 interface. These microscopic features align with the large entropy gain detected experimentally, suggesting a mechanistic link between structural plasticity and thermodynamic favorability.

## 4. Materials and Methods

### 4.1. Investigated Peptides

All four peptides were synthesized by KareBay Biochem (Monmouth Junction, NJ, USA) and showed a purity of 98% and more. The human ACE2 protein (hACE2) was purchased from AbClonal (Woburn, MA, USA) (catalogue no: RP01277). It is a recombinant, His-tagged protein consisting of Gln18-Ser740 from human ACE2 (accession number Q9BYF1) and was produced in the HEK293 cell expression system. According to the manufacturer, the protein has a purity of over 95% as determined by SDS/PAGE. PBS buffer was purchased from Sigma-Aldrich (St. Louis, MO, USA), and NaOH and NaCl were purchased from Chempur (Piekary Slaskie, Poland). All reagents were of analytical grade. Deionized water with a conductivity of no more than 0.05 μS/cm was used for the preparation of all aqueous solutions.

### 4.2. Dialysis and ITC Experiments

Isothermal titration calorimetry measurements were performed at 25 °C and a pH of 7.4 on a MicroCal PEAQ Isothermal Titration Calorimeter (Malvern Panalytical Ltd., Malvern, UK). PBS buffer (Merck KGaA, Darmstadt, Germany) was prepared from tablets, and the pH was adjusted using HCl and NaOH. After the device was stabilized at 25 °C, 40 μL of buffered peptide solutions was used to titrate 200 μL of buffered ACE2 solutions (the concentration was initially about 10 times lower than that of RBD) by 19 consecutive injections with an interval of 150 s between each drop and a stirring speed of 750 rpm (each test was repeated a few times). The reference cell was filled with deionized water. Data were fitted using MicroCal PEAQ-ITC analysis software. An initial injection of 0.4 μL was subtracted from each data set to eliminate the effect of diffusion of the titrant through the syringe tip during the equilibration process. The thermodynamic parameters (binding affinities, enthalpy and entropy changes) were determined by a combination of nonlinear least squares fitting and the selection of an appropriate model to describe the studied binding interaction [40]. A CaCl_2_-EDTA titration was performed to check the instrument, and the results were compared with those obtained for the same samples (test kit) using MicroCal. The heat of dilution was subtracted from each injection [41]. All ITC studies were performed after extensive dialysis of the protein against 1 L of PBS buffer at 5 °C. For each ITC assay, the hACE2 receptor protein was dialyzed against the same buffer and for the same time period (the buffer was replaced 4 times every 12 h) to ensure that all samples were as pure as possible and fitted into the correct buffer to avoid heat changes due to buffer mismatch. The concentration of each protein portion was determined by measuring the UV absorbance at 280 nm using the NanoDrop One C spectrophotometer (Waltham, MA, USA). The theoretical extinction coefficient for ACE2 (calculated with Expasy ProtParam) was 169,180 M^−1^cm^−1^. The ITC measurements were performed immediately after dialysis and the peptides were dissolved in the buffer of the last dialysis of the ACE2 protein.

### 4.3. Input Models for the MD Simulations

The PDB ID 1R42 [42] structure was employed as a starting model of the ACE2 receptor. This structure was chosen among the 248 available experimental structures because it is the highest resolution structure of the native, unbound receptor. The structure contains the positions of the zinc metallopeptidase domain (residues 19–611) and a few residues of the collectrin-like domains (612–615) of the extracellular region of ACE2. ACE2 is a glycoprotein whose glycosylation affects SARS-CoV-2 S protein binding [22]. We added glycans to the 1R42 model following the findings in [43] (Supplementary Figure S4) using the Glycan Reader and Modeler [44] module of the CHARMM-GUI server [45].

In the spike–ACE2 complex, the RBM adopts an extended and somewhat disordered conformation, which can, therefore, be used to derive a reasonable starting model of the free-state conformations of the investigated peptides. Specifically, for the development of the peptide models, the segment of interest (aa G^482^–G^502^) was cut off the SARS-CoV-2 Spike protein–ACE2 complex structure with the PDB ID 6M0J [9]. The N-terminus of the fragment was acetylated, a free amine group was added to its C-terminus, and the structure was equilibrated within a 50 ns equilibration MD simulation. The final configuration was used as the starting model of the WT-SARS-CoV-2 peptide fragment. To ensure the same initial conditions for all four variants, the amino acid residues at positions 484 and 501 of this WT fragment structure were mutated accordingly (Figure 1) without changing the backbone torsional angles of these two residues using the PyMOL package [46] to develop the models of the Alpha, Beta/Gamma, and Zeta variant peptides. The models were equilibrated using 50 ns MD simulations in which peptides were allowed to freely explore their conformational space.

### 4.4. Molecular Dynamics Simulation Protocol

The peptides were initially placed randomly around the receptor protein at a distance of approximately 2 nm. This distance was chosen as a compromise between the simulation box size (and the corresponding computational resources) and the cutoff range for long-range interactions in the system. It allows the peptides to move freely around the receptor, ensuring that the binding process is not biased toward a specific binding site on ACE2. This setup closely mimics the experimental conditions of the ITC measurements, where the two binding molecules are free in solution and can interact without spatial constraints.

The MD simulations were performed using GROMACS 2022.3 [47]. The CHARMM36 force field was chosen to parameterize the glycosylated protein and the peptide fragments [48,49]. The peptides were randomly positioned around the receptor protein at an initial distance of about 2 nm. The systems were solvated in dodecahedral boxes with a minimal distance between the solute and the box of 1.5 nm. After neutralization with counter-ions and the addition of 0.15 mM NaCl to mimic physiological conditions, energy minimization was performed to relax any steric clashes or unfavorable contacts within the system until the maximum force on any atom was less than 100 kJ/(mol·nm). The systems were then subjected to a 400 ps equilibration simulation under NPT conditions at T = 310 K and *p* = 1 atm, with positional restraints applied to the heavy atoms of the proteins. For the production runs, the systems were maintained at 310 K and 1 atm using the V-rescale thermostat [50] and Parrinello–Rahman barostat [51]. The coupling constants for temperature and pressure were set to 0.1 ps and 2.0 ps, respectively. Long-range electrostatic interactions were computed using the fast smooth particle-mesh Ewald [52] method with a direct space cutoff of 1.2 nm. Van der Waals forces were smoothly switched off from 1.0 to 1.2 nm. Covalent bonds between heavy and hydrogen atoms were constrained using P-LINCS [53] to allow for a 2 fs time step of the leap-frog integrator [54]. For each peptide variant, two independent 400-nanosecond simulations were conducted, starting from different initial configurations of the fragment, amounting to 800 ns total simulation time per peptide.

### 4.5. Synthetic Data Analysis

The MD data were analyzed using the built-in GROMACS post-processing and analysis tools [55]. Structure visualizations were generated using the molecular visualization and manipulation package VMD [56]. Schematic diagrams of the ACE2–peptide interactions were obtained with the LigPlot+ program [57].

### 4.6. Estimation of the Binding Free Energy

A convolutional neural network was designed to predict the binding free energy of protein–peptide complexes. The network processes a 3D spatial grid of dimensions 80 × 80 × 80 × 1, where each voxel represents a spatial unit within the domain. The central position of each amino acid residue in the complex is assigned a categorical value, distinguishing between 20 receptor residue categories and 20 ligand residue categories, ensuring clear differentiation between the two components.

The architecture consists of two parallel convolutional pathways to extract hierarchical spatial features. Each pathway begins with an initial 3D convolutional layer with 20 filters and a (2, 2, 2) kernel size, followed by a pooling layer to reduce spatial dimensions and enhance feature abstraction. The first pathway applies max-pooling, while the second uses average-pooling, capturing complementary spatial information. Both pathways proceed through five additional 3D convolutional layers, each with 10 filters, to extract increasingly complex spatial patterns. A second pooling layer is applied at the end of each pathway to further refine the learned representations.

The outputs from both pathways are flattened and concatenated, integrating features extracted through both pooling strategies. This combined feature vector is processed through a series of fully connected (dense) layers, with progressively decreasing units (8, 4, 2) to improve generalization and minimize overfitting. The final dense layer outputs a single scalar value representing the predicted binding free energy. The network is trained using the Adam optimizer, minimizing mean squared error (MSE), with mean absolute error (MAE) as an additional evaluation metric.

This dual-pathway CNN architecture ensures comprehensive feature extraction, leveraging both max-pooling and average-pooling strategies to capture diverse spatial characteristics essential for accurate binding free energy prediction. The CNN was trained and validated using 183 peptide/protein complexes from the PROXiMATE database [58]. The training set consisted of 150 complexes, while 33 complexes were used for validation. The model achieved a validation loss of 7.09 (kcal/mol)^2^ and a mean absolute error (MAE) of 1.93 kcal/mol.

## 5. Conclusions

In this study, we investigate the impact of the N501Y and E484K mutations in the SARS-CoV-2 receptor-binding motif (RBM) on ACE2 binding affinity through isothermal titration calorimetry (ITC) and molecular dynamics (MD) simulations.

The ITC measurements revealed that both mutations influence RBM-ACE2 binding affinity, with the N501Y mutation, characteristic of the Alpha variant, increasing the affinity by 2.2-fold, and the E484K mutation, associated with the Zeta variant, resulting in a 5.8-fold enhancement. Notably, the RBM fragment containing both mutations, as seen in the Beta and Gamma variants, exhibited the strongest binding, with a 15-fold increase in affinity.

Thermodynamic analysis indicated that the binding interaction for the Zeta peptide (E484K mutation) is predominantly enthalpy-driven. In contrast, the Beta/Gamma peptide (containing both N501Y and E484K mutations) exhibited a strong entropic contribution, implying that the combined mutations induce conformational changes or alter the dynamics of the RBM, potentially increasing hydrophobic interactions and reducing structural rigidity upon binding.

MD simulations confirmed that all RBM peptides target the ACE2 domain responsible for spike binding, albeit with distinct binding poses. The Beta/Gamma peptide interacts with a secondary site (K^313^–N^321^, H^417^–I^421^, T^548^–R^559^, plus glycosylation sites at N^90^, N^322^, and N^546^), while the Zeta peptide intermittently engages a nearby site (T^324^, Q^325^, K^353^–R^357^, A^386^–A^387^, N^556^–V^574^, and the same glycans). A strong correlation was observed between the entropic contribution and the number of hydrophobic contacts: the Beta/Gamma peptide formed 18 such contacts, yielding the highest entropy gain. Binding enthalpy did not correlate with hydrogen bond count unless glycan interactions were included, which markedly improved the correlation.

Relative changes in binding affinity (ΔK_D_) and binding free energy (ΔG) were calculated to minimize the impact of experimental variations, primarily stemming from differences in RBD construct length and measurement methodologies. Our analysis confirmed that the N501Y mutation enhances ACE2 binding, while the effect of E484K remains uncertain, as its binding energy is statistically comparable to that of the wild type. Notably, theoretical ΔG predictions consistently overestimate experimental values, with only the Prodigy server providing estimations that are reasonably close to experimental data, whereas MD-based free energy calculations show significant deviations. Additionally, our CNN model predictions align closely with those from the Prodigy server, further supporting the observed binding affinity trends for the studied mutations.

Our findings underscore the complex interplay between different mutations in the SARS-CoV-2 spike protein and their collective impact on the thermodynamics of RBM-ACE2 interactions. The entropy-driven binding in Beta/Gamma variants, along with the identification of secondary binding sites, suggests that viral mutations not only enhance affinity but also introduce alternative receptor interactions. These findings provide deeper insight into SARS-CoV-2 evolution, particularly regarding transmissibility and potential therapeutic strategies. Future studies should further explore glycosylation effects and alternative binding sites, as well as investigate the impact of these mutations on immune escape mechanisms.

## Figures and Tables

**Table 1:** The thermodynamic parameters of the interactions between hACE2 receptor and RBM fragments, measured at pH 7.4 and 25 °C. The estimated standard deviations were calculated based on at least two independent experiments.

RBM Fragments	K_D(ITC)_ [μM]	N	ΔG_ITC_ [kcal/mol]	ΔH_ITC_ [kcal/mol]	−TΔS_ITC_ [kcal/mol]
WT	4.74 ± 1.45	0.96 ± 0.13	−7.33 ± 0.31	−3.40 ± 0.68	−3.94 ± 0.75
Alpha	2.15 ± 0.83	0.96 ± 0.14	−7.74 ± 0.39	−3.29 ± 0.70	−4.44 ± 0.80
Beta/Gamma	0.31 ± 0.08	0.91 ± 0.02	−8.89 ± 0.26	−2.28 ± 0.09	−6.61 ± 0.27
Zeta	0.81 ± 0.06	0.76 ± 0.03	−8.31 ± 0.07	−4.26 ± 0.24	−4.06 ± 0.25

## Data Availability

The additional data supporting the manuscript are available from the corresponding authors upon reasonable request.

## Supplementary Materials

The following supporting information can be downloaded at https://www.mdpi.com/article/10.3390/ijms26094064/s1.

## Abbreviations

The following abbreviations are used in this manuscript:

ACE2	Angiotensin-converting enzyme 2
BLI	Biolayer interferometry
CNN	Convolutional neural network
ΔG	Binding free energy
ΔH	Binding enthalpy
ITC	Isothermal titration calorimetry
K_D_	Dissociation constant
MD	Molecular dynamics
RBD	Receptor-binding domain
RBM	Receptor-binding motif
SARS-CoV-2	Severe acute respiratory syndrome coronavirus 2
SPR	Surface plasmon resonance
TΔS	Entropic contribution to the binding free energy
VOC	Variants of concern
VOI	Variants of interest
WHO	World Health Organization
WT	Wild type
