# Supplementary material for: Impact of SARS-CoV-2 RBM Mutations N501Y and E484K on ACE2 Binding: A Combined Computational and Experimental Study
Source: Int J Mol Sci. 2025 Apr 25;26(9):4064. doi: 10.3390/ijms26094064 (PMC12071706; doi:10.3390/ijms26094064)
Supplement: Supplementary file 1 [file ijms-26-04064-s001.zip › ijms-3568482-supplementary.pdf]

Supplementary Materials: Impact of SARS-CoV-2 RBM Mutations N501Y and E484K on ACE2 Binding: A Combined Computational and Experimental Study

Agnieszka Rombel-Bryzek*, Peicho Petkov*, Elena Lilkova, Nevena Ilieva, Leandar Litov, Mariusz Kubus, and Danuta Witkowska

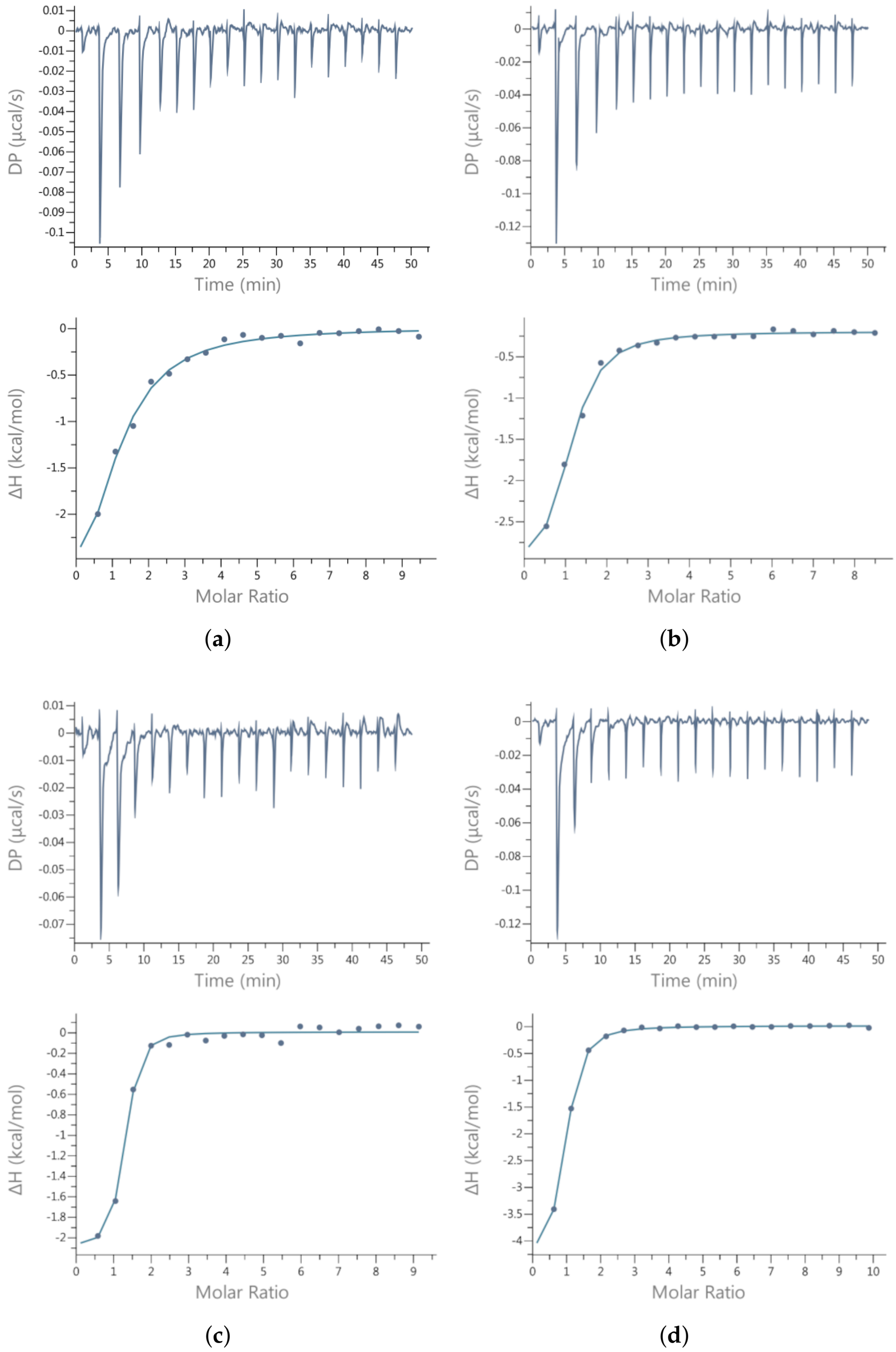

Figure S1. ITC curves. Binding thermodynamics to ACE2 of the (**a**) WT, (**b**) alpha, (**c**) beta/gamma, and (**d**) zeta RBM peptide fragments.

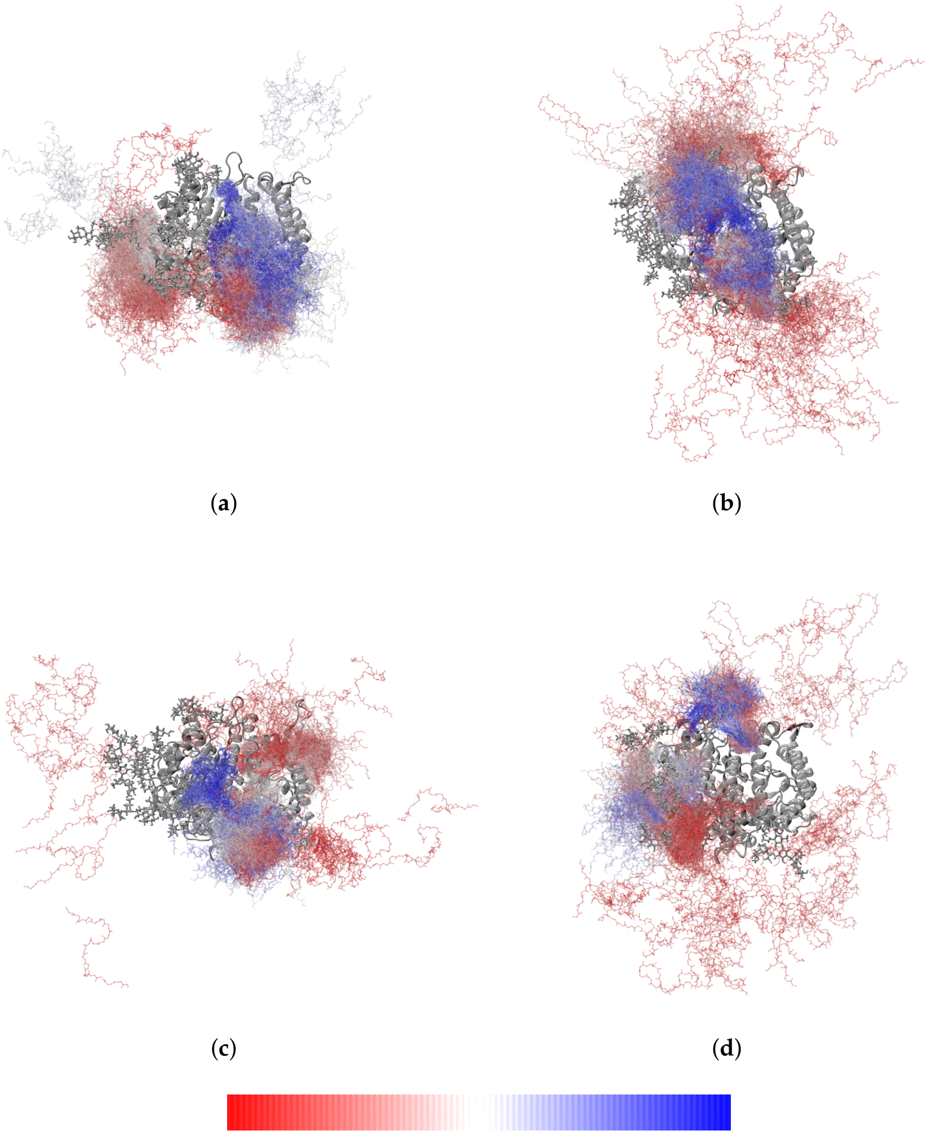

(**a**) (**b**)

(**c**) (**d**)

(**e**)

Figure S2. MD generated conformational ensembles of the (**a**) WT; (**b**) alpha; (**c**) beta/gamma; and (**d**) zeta RBM peptides around the glycosylated hACE2 receptor. (**e**) Peptide conformations are coloured by timestep from red to blue. For better visibility only the peptide backbone positions are depicted with linesevery 250 ps.

Table S1. Summary of experimental studies of the the E484K and/or N501Y mutations effect on the SARS-CoV-2 RBD — hACE2 receptor dissociation constant K_D.

Pub.	Ref.	Method	Experimental details
1	[1]	SPR	T=37°C, buffer – 25 mM NaH_2PO_4 and 150 mM NaCl at pH 7.5, RBD (spike aa 319–541) with a C-terminal oligohistidine tag.
2	[2]	SPR	T=25°C, buffer – 25 mM Hepes-KOH pH 7.5, 150 mM NaCl, 10 µM ZnCl2 to stabilize hACE2, 0.05% Tween 20; RBD (spike aa 333–529)
3	[3]	SPR	buffer – 0.15 M NaCl, 20 mM HEPES, pH 7.4 ;RBD (spike aa 319–591)
4	[4]	SPR	T=25°C, buffer – 0.01 M HEPES, 0.15 M NaCl, 0.003 M EDTA, and 0.05% (V/V) surfactant P20, pH 7.4; RBD
5	[5]	BLI	kinetics buffer; RBD
6	[6]	MST	room T; RBD
7	[7]	ITC	T=25°C; buffer – PBS pH 7.4; RBD (spike aa 319–541) fused with a hexahistidine tag
8	[7]	BLI	T=25°C; RBD (spike aa 319–541) fused with a hexahistidine tag
9	[8]	BLI	HIS-tagged RDB
10	[9]	BLI	RBD (spike aa 319–593)
11	[10]	BLI	buffer – PBS, 0.1% BSA, 0.02% Tween 20; RBD (spike aa 319–541) fused with a hexahistidine tag
12	here	ITC	T=25°C, buffer – PBS The PBS buffer, pH =7.4 ; RBM fragment (spike aa 482–502)

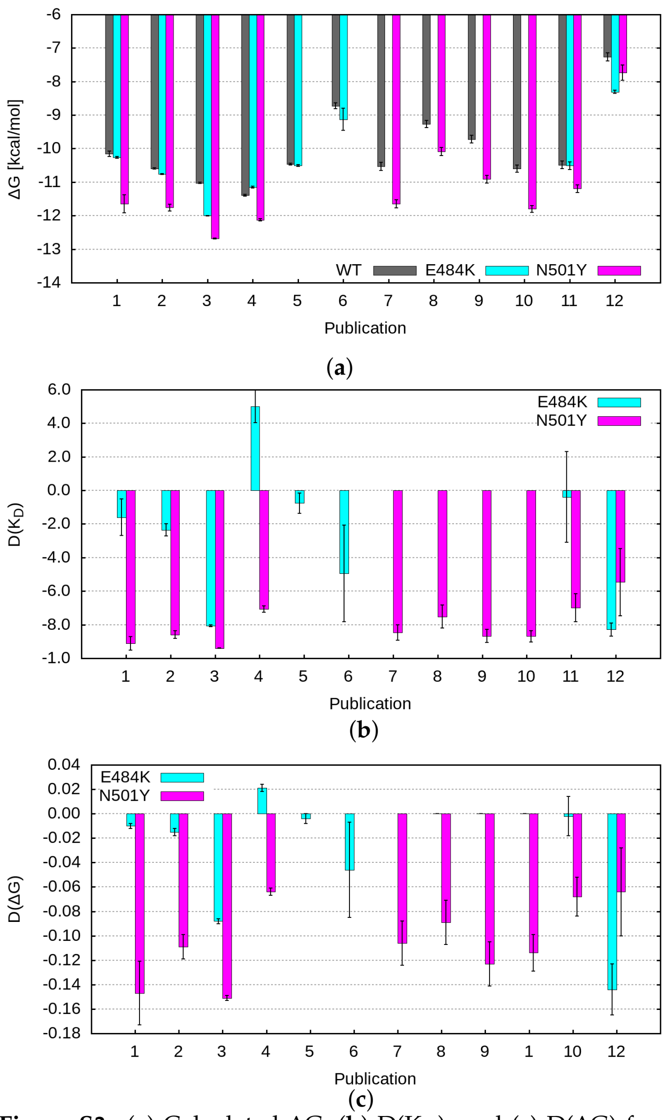

Figure S3. (**a**) Calculated ΔG; (**b**) D(K_D); and (**c**) D(ΔG) for the WT and the E484K and N501Y mutations. The dots on the box diagram correspond to our data. Data is based on experimental findings in the publications listed in Suppl. Table S1.

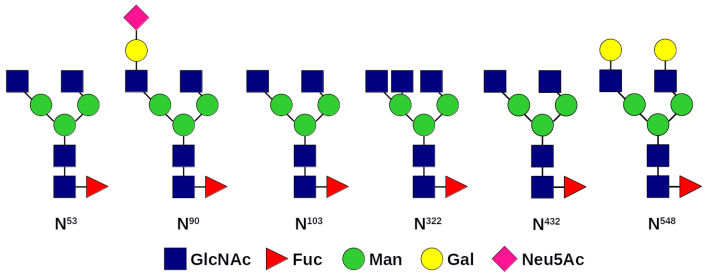

Figure S4. Glycosylation of the ACE2 receptor model.

References

1. Barton, M.; MacGowan, S.; Kutuzov, M.; Dushek, O.; Barton, G.; Van Der Merwe, P. Effects of common mutations in the SARS-CoV-2 Spike RBD and its ligand, the human ACE2 receptor on binding affinity and kinetics. *eLife* **2021**, *10*, e70658. https://doi.org/10.7554/eLife.70658.
2. Laffeber, C.; De Koning, K.; Kanaar, R.; Lebbink, J. Experimental Evidence for Enhanced Receptor Binding by Rapidly Spreading SARS-CoV-2 Variants. *Journal of Molecular Biology* **2021**, *433*, 167058. https://doi.org/10.1016/j.jmb.2021.167058.

3. Tian, F.; Tong, B.; Sun, L.; Shi, S.; Zheng, B.; Wang, Z.; Dong, X.; Zheng, P. N501Y mutation of spike protein in SARS-CoV-2 strengthens its binding to receptor ACE2. *eLife* **2021**, *10*, e69091. https://doi.org/10.7554/eLife.69091.
4. Raghu, D.; Hamill, P.; Banaji, A.; McLaren, A.; Hsu, Y. Assessment of the binding interactions of SARS-CoV-2 spike glycoprotein variants. *Journal of Pharmaceutical Analysis* **2022**, *12*, 58–64. https://doi.org/10.1016/j.jpha.2021.09.006.
5. Augusto, G.; Mohsen, M.; Zinkhan, S.; Liu, X.; Vogel, M.; Bachmann, M. In vitro data suggest that Indian delta variant B.1.617 of SARS-CoV-2 escapes neutralization by both receptor affinity and immune evasion. *Allergy* **2022**, *77*, 111–117. https://doi.org/10.1111/all.15065.
6. Ramanathan, M.; Ferguson, I.; Miao, W.; Khavari, P. SARS-CoV-2 B.1.1.7 and B.1.351 Spike variants bind human ACE2 with increased affinity, 2021. Available at: http://biorxiv.org/lookup/doi/10.1101/2021.02.22.432359.
7. Prévost, J.; Richard, J.; Gasser, R.; Ding, S.; Fage, C.; Anand, S.P.; Adam, D.; Gupta Vergara, N.; Tauzin, A.; Benlarbi, M.; et al. Impact of temperature on the affinity of SARS-CoV-2 Spike glycoprotein for host ACE2. *Journal of Biological Chemistry* **2021**, *297*, 101151. https://doi.org/10.1016/j.jbc.2021.101151.
8. Supasa, P.; Zhou, D.; Dejnirattisai, W.; Liu, C.; Mentzer, A.J.; Ginn, H.M.; Zhao, Y.; Duyvesteyn, H.M.; Nutalai, R.; Tuekprakhon, A.; et al. Reduced neutralization of SARS-CoV-2 B.1.1.7 variant by convalescent and vaccine sera. *Cell* **2021**, *184*, 2201–2211.e7. https://doi.org/10.1016/j.cell.2021.02.033.
9. Bayarri-Olmos, R.; Johnsen, L.B.; Idorn, M.; Reinert, L.S.; Rosbjerg, A.; Vang, S.; Hansen, C.B.; Helgstrand, C.; Bjelke, J.R.; Bak-Thomsen, T.; et al. The alpha/B.1.1.7 SARS-CoV-2 variant exhibits significantly higher affinity for ACE-2 and requires lower inoculation doses to cause disease in K18-hACE2 mice. *eLife* **2021**, *10*, e70002. https://doi.org/10.7554/eLife.70002.
10. Vogel, M.; Augusto, G.; Chang, X.; Liu, X.; Speiser, D.; Mohsen, M.O.; Bachmann, M.F. Molecular definition of severe acute respiratory syndrome coronavirus 2 receptor-binding domain mutations: Receptor affinity versus neutralization of receptor interaction. *Allergy* **2022**, *77*, 143–149. https://doi.org/10.1111/all.15002.

Disclaimer/Publisher's Note: The statements, opinions and data contained in all publications are solely those of the individual author(s) and contributor(s) and not of MDPI and/or the editor(s). MDPI and/or the editor(s) disclaim responsibility for any injury to people or property resulting from any ideas, methods, instructions or products referred to in the content.
